# Desiccant dust and the use of CO_2_ gas as a mobility stimulant for bed bugs: a potential control solution?

**DOI:** 10.1007/s10340-016-0784-1

**Published:** 2016-06-20

**Authors:** Anders Aak, Espen Roligheten, Bjørn Arne Rukke, Tone Birkemoe

**Affiliations:** 1 Department of Pest Control, Norwegian Institute of Public Health, Lovisenberggata 8, 4404 Nydalen, NO-0456 Oslo, Norway; 2 Oslo Boligbygg, Wergelandsveien 3, Postboks 1192, Sentrum 0107 Oslo, Norway; 3 Ecology and Natural Resource Management. Campus Ås, Norwegian University of Life Sciences, Universitetstunet 3, NO1430-Ås Oslo, Norway

**Keywords:** *Cimex lectularius*, Desiccant dust, Mortality, Olfaction, Pest, CO_2_

## Abstract

The common bed bug (*Cimex lectularius,* Hemiptera; Cimicidae) infests homes and service industries, and the number of infestations has greatly increased over the past 20 years. At present, no cost-effective control methods are available, and eradication programs are expensive and laborious. We investigated the control potential of desiccant dust in combination with CO_2_ as a bed bug activity stimulant. An initial experiment with two desiccant dusts was followed by arena studies with varying doses, available hiding places and the presence or absence of host signals. Finally, we conducted a field experiment with Syloid 244FP with or without CO_2_ gas. Syloid was superior compared to diatomaceous earth, and effective at the concentration of 1.0 g/m^2^ in the field experiment. The number of harborages and partial application of desiccant dust decreased mortality in the laboratory. Bed bug activation by CO_2_ appeared of minor importance in the arena studies, but was crucial for the eradication in the student dormitories. In fact, all 5 bed bug-infested dormitories with a combined treatment of desiccant dust and CO_2_ were freed of bed bugs, whereas eradication was not successful in any of the 6 dormitories with only desiccant dust treatment. The different results in the laboratory and field experiment were most likely caused by the longer activation and higher dose of CO_2_ used in the field experiment than the laboratory experiment. Our study showed that application of desiccant dust in combination with release of CO_2_ gas to mimic human presence is a promising option for bed bug control.

## Key message

- Cost-effective control methods are currently not available to deal with the increasing bed bug problems.
- Desiccant dusts were evaluated for control efficacy in dose–response experiments and arena bioassays that simulated infested rooms.
- Availability of bed bug harborages and application rates of the desiccant dust affected bed bug survivorship, and CO_2_ activation can be utilized in an attract-and-kill solution to improve field efficacy.
- The use of CO_2_ gas to mimic human presence in combination with desiccant dust was found to be a promising option for bed bug control.

## Introduction

The common bed bug, *Cimex lectularius* (Hemiptera; Cimicidae), has made a major comeback as a nuisance pest during the last 20 years, affecting private homes and the commodity industry (Doggett et al. 2012). Effective pesticides are no longer available due to resistance and increased awareness of human health safety (Davies et al. 2012). At present, a wide array of management methods are incorporated in integrated pest management solutions which in most cases are labor intensive and financially costly in general. Consequently, the development of cost- and labor-effective methods is strongly needed (Weeks et al. 2010; Doggett et al. 2012; Koganemaru and Miller 2013).

Central life history elements are often targeted for insect pest population decimation (Stearns 1992; Dent 2000; Minakuchi and Riddiford 2006; Witzgall et al. 2010), but in bed bug control they are hard to impact. Mating, egg laying, and molting take place inside hidden harborages, and there is a dynamic exchange and dispersal of individuals between already established and new harborages (Cooper et al. 2015). To have a successful eradication of bed bugs, most hiding places needs to be found and reached with effective insecticides. Most bed bug activity also occurs at night, and adults are able to hide in harborages as well as in small cracks and crevices outside of harborages during the day. However, bed bugs depend on their host for food and have an innate and strong response to host signals (Reinhardt and Siva-Jothy 2007). CO_2_, for instance, makes hungry bed bugs leave their harborages to initiate oriented movement to locate their blood meal (Suchy and Lewis 2011; Aak et al. 2014; Singh et al. 2015a). When bed bugs detect the CO_2_, the host cue, they come outside from their harborages to feed; during which they may potentially be exposed to insecticides. Thus, bed bug control by using the CO_2_ to imitate the presence of human host may help the insecticide to reach the bed bugs without identifying or localizing all hiding places.

With the exception of three studies (Feldlaufer and Blomquist 2011; Koganemaru et al. 2013; Lilly et al. 2016), details of the bed bug integument structuring are poorly described. However, in relation to desiccant dust functionality, several observations show that an effective disruption of the cuticle function by removal of lipids leads to increased mortality due to desiccation (Benoit et al. 2009b; Anderson and Cowles 2012; Akhtar and Isman 2013; Akhtar and Isman 2016). Application of desiccant dust supported by other methods indicates field mortality (Wang et al. 2009a; Wang et al. 2013) and products and effectiveness reports are emerging in pest control magazines (Potter et al. 2013; Potter et al. 2014). However, comprehensive scientific field evaluations identifying the pure effect from desiccants are lacking. When applied to control other pest insects, the desiccation effect is reduced if the environment is moist and the chance of coming in contact with the insecticidal dust is low if insects have the option to move outside of dust-treated areas (Ebeling 1971; Shah and Khan 2014). These problems may be of less consequence in bed bug control. The bed bugs are often found in discrete indoor environments, typically single rooms or apartments, and it is possible to reach most areas in need of treatment. The use of dust as a killing agent may thus have a potential not yet fully recognized in the control of bed bugs.

The aim of this study was to test the combined effect of various CO_2_ concentrations in the environment and dust exposure on bed bug survivorship. We used a three-step approach to investigate the potential use of combined insecticidal dust and CO_2_ in bed bug eradication programs: 1) dose–response experiments of two different desiccant dusts, 2) arena studies with desiccant dust and varying number of potential new harborages with and without changing the CO_2_ concentration, and 3) field experiments with desiccant dust only or desiccant dust combined with various CO_2_ concentrations in infested student dormitories.

## Materials and methods

### Insect colony rearing and preparation of experimental individuals

Prior to all experiments, bed bugs were kept in 140-ml polyethylene rearing boxes (VWR straight sample container, VWR, Oslo, Norway) in climate chambers (Sanyo - MLR-351H, Medinor ASA, Oslo, Norway) under a photoperiod of 16:8 (light:dark) hours at 22 °C and 65 % relative humidity (RH). The plastic lids of the boxes had circular openings (40-mm diameter) in which metal mesh screens (0.25 mm openings; Burmeister AS, Oslo, Norway) were inserted to facilitate the passage of air and to allow bed bugs to feed. The bed bugs were fed with heated human blood through a Parafilm membrane (Aak and Rukke 2014). Bed bugs in rearing boxes were collected from two hotels in Oslo, Norway, in 2009. For the experiments, fifth-instar nymphs were selected from the rearing boxes, transferred to a new box, and fed. Adults used in the experiments emerged in these rearing boxes less than 14 days prior to the start of the experiment.

### Dose–response of desiccant dusts

We tested the efficacy of two types of desiccant dusts: Syloid 244 FP (GRACE GmbH & CO, Germany—hereafter denoted as *Syloid*) and Myrnix (Tergent AB, Sweden—hereafter denoted as *diatomaceous earth*). Syloid is synthetic amorphous silica powder (99.6 % SiO_2_) with particle size of 5.5 µm and Myrnix is naturally occurring remains of fresh water hard-shelled algae (99–100 % SiO2) with a particle size of 2–18 µm. Syloid is used as excipients in many pharmaceutical formulations and Myrnix is a registered pesticide with recommended application rates of 10–20 g/m^2^. Both desiccant dusts were tested at four different concentrations of 3.0, 1.0, 0.3, and 0.1 g/m^2^. A filter paper was placed inside a Petri dish with 90 mm diameter, desiccant dust was applied, and bed bugs were released on the filter paper inside the Petri dish. A total of 216 bed bugs were used and the sex ratio was balanced between each treatment. Twenty-four adult bed bugs were used per desiccant dust concentration. For practical reasons (ease of handling and counting), they were distributed in 6 Petri dishes (90 mm in diameter) with 4 individuals in each. The effect was compared to control Petri dishes without the desiccant dust. The dust was weighed to 0.001 g precision on an analytical balance (Sartorius BP211D; Sartorius AG, Göttingen, Germany) before being evenly distributed on a filter paper in the Petri dishes. Bed bugs were fed to repletion 24 h before being transferred to the Petri dishes, and mortality was recorded every 24 h for 7 days. The ambient temperature and humidity during the experiment were 22–24 °C and 11–36 % RH, recorded using Tiny Tag data loggers (Presisjonsteknik, Oslo, Norway). The experiment was performed in a laboratory following the natural daylight period of 15:9 h of day:night.

### Arena experiments

**Fig. 1:**
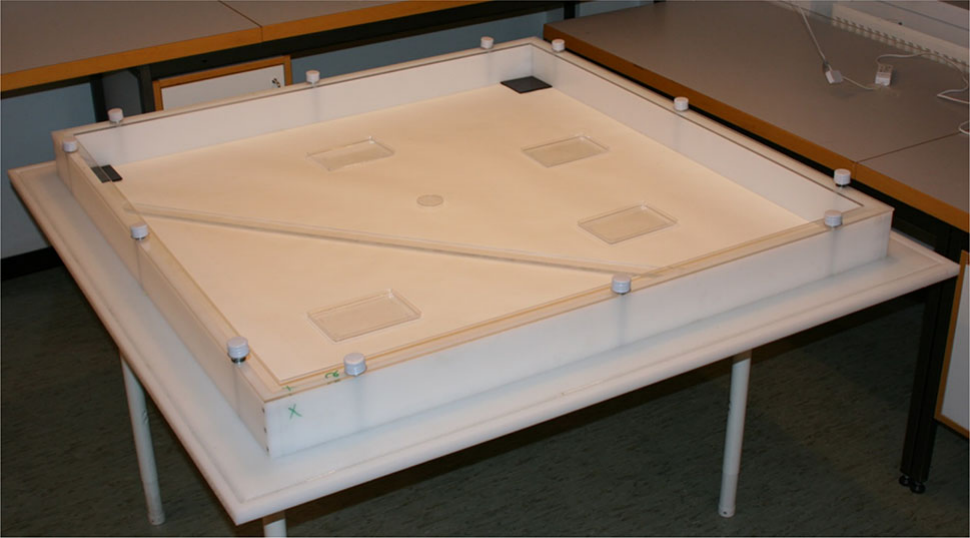
A bed bug arena used to investigate the effect of different Syloid treatments. Harborages are located in each of the four corners of the arena and transparent plastic plates provided additional possible new harborages. The inverted Petri dish in the center was used as an elevated platform to place dry ice that release CO_2_ gas for stimulating the bed bugs

To simulate the field environment in a controllable setting we used arenas measuring 126 × 126 cm to represent an infested room (Fig. 1). Three identical arenas were used simultaneously in one large laboratory room. The arenas followed a previously described design in terms of bed bug traveling space, floor substrate, light positioning, placement of harborages, escape barriers, and camera (Aak et al. 2014). In these three arenas, the Plexiglas walls of the previously described arena design were replaced with white, 2-cm thick plastic walls. No inner frame was necessary as the walls weighed down and kept the paper on the arena floor in place. An inverted Petri dish (5.5 cm) was placed in the middle of each arena and used as an elevated platform to place dry ice to release the CO_2_ gas to stimulate and attract the bed bugs. The room had a photoperiod of 16:8 (light:dark) hours, which was synchronized with the climate chambers. The temperature and humidity during the experiment was 22–23 °C and 14–40 % RH, recorded using Tiny Tag data loggers. Syloid was used as the desiccant dust treatment as it proved superior in the dose–response experiment.

Prior to the experiment, bed bugs were blood fed to repletion and then kept in the climate chambers for three days. After these three days, 10 bed bugs were released in the open spaces of the arena and given another three days to acclimate to the environment and the harborages. Desiccant dust was then applied and distributed evenly in the arena using a double sieve (Endecotts Ltd., London, England) with the first sieve having an aperture length of 1.0 mm followed by a second sieve with aperture length of 0.5 mm. The edges of the arena were covered with a 13-cm-wide paper strip to provide the bed bugs with an untreated space along the walls. Bed bugs found outside harborages at application of the desiccant dust, were covered with an inverted Petri dish to avoid direct dust exposure. To simulate the field setting during desiccant dust treatment in an infested room, dust and bed bugs were kept in the arena for 4 days in all experiments with the exception of the partly treated arena described below. In the stimulation period, a Petri dish was introduced in the center of the arena, and dry ice was placed on the Petri dish as a CO_2_ source. The dry ice was weighed before and after use to quantify the amount released. The mean (±SE) release rate of CO_2_ was 360 ± 0.02 mg/min (range: 150–450 mg/min). On a daily basis and 9 h into the photophase, the CO_2_ stimulant or an empty Petri dish was introduced and left there for 30 min. Between each experimental replicate, the arenas were cleaned by vacuuming thoroughly two times and wiping the floor and walls twice using microfiber dusting cloths (JIF‐tørrmopp, Lilleborg AS, Norway) to remove all desiccant dust.

We performed four experiments consisting of 6 or 12 arena replicates in addition to 9 control replicates (Table 1). All replicates contained 10 adult bed bugs in each arena. The male:female ratio was always 1:1. Two measurements were taken to evaluate the results: mortality and video-recorded bed bug movement during the CO_2_ gas release. At the same time as the Petri dishes were introduced, daily mortality was scored by visually inspecting the bed bugs. At the end of each dust treatment, the bed bugs were collected from the arenas, and survivors were put into empty polyethylene rearing boxes with a 2 × 2 cm piece of filter paper (VWR qualitative filter paper, VWR, Oslo, Norway). The bed bugs were then placed in the climate chambers and mortality was recorded every day for 10 days. Bed bug movement was detected using a video camera in the control, high dose and the partially applied dose experiments described below. Movement was scored using the accumulated number of individuals moving within one minute. These numbers were then averaged across each 5-min period to be used in the statistical analyses. Cameras recorded movement for 15 min before introduction of CO_2_, for 30 min during CO_2_ release and for 30 min after dry ice removal.

**Table 1:** An overview of the arena experiments conducted. All replicates consisted of 10 adults of *Cimex lectularius*

	Desiccant dust treatment				
	Control	High dose	Low dose	Low dose + many hiding places	Low dose, partly applied + many hiding palaces
Replicates with CO_2_	6	3	6	6	6
Replicates without CO_2_	3	3	6	6	6
Application rate	0.0 g/m^2^	3.0 g/m^2^	0.3 g/m^2^	0.3 g/m^2^	0.3 g/m^2^
Applied area	–	In whole arena	In whole arena	In whole arena	25 % of the arena
Exposure time	4 days	4 days	4 days	4 days	2 days
Harborages	4 harborages *Or* 4 harborages 1 rod 4 plates	4 harborages	4 harborages	4 harborages 1 rod 4 plates	4 harborages 1 rod 4 plates
*Measurements*					
Mortality	Yes	Yes	Yes	Yes	Yes
Activity observed	Yes	Yes	No	No	Yes

#### High dose

Syloid dust was applied 3.0 g/m^2^ to the arena containing four harborages (Fig. 4a).

#### Low dose

Syloid dust was applied 0.3 g/m^2^ to the arena containing four harborages (Fig. 4b).

#### Low dose and additional harborages

Syloid dust was applied 0.3 g/m^2^ to the arena containing four harborages. To simulate clutter, cracks and other environmental structures, we also added a 130-cm-long and 4-cm-wide Plexiglas rod and four 11 × 18 cm transparent plastic plates to the arenas before releasing the bed bugs (Fig. 4c). The rod was positioned flat on the arena floor, running diagonally and approximately 15 cm to one side of the center of the arena. The plates were distributed randomly in the remaining open space (Fig. 1). Two corners of the rod and one corner of each plate were slightly elevated with tiny plastic capsules underneath them. This allowed bed bugs to hide underneath the objects.

#### Low dose, partly applied, and many hiding places

Syloid dust was applied 0.3 g/m^2^ only in the center 0.25 m^2^ of the arena containing four harborages and additional harborages (Fig. 4d). The treatment was terminated after 2 days instead of 4, reflecting average days of room vacancy at hotels.

#### Control

To ensure that the arena and the stimulant did not induce mortality, bed bugs were tested in cleaned arenas with one harborage in each of the four corners with or without CO_2_ stimulation and in cleaned arenas with new potential harborages combined with CO_2_ stimulation (Fig. 4e).

### Field experiments

Eleven bed bug-infested rooms and apartments located in two large student complexes in the counties of Oslo and Akershus, Norway, were used in this experiment. Syloid was used as the desiccant dust treatment as it proved superior in the dose–response experiment. Only rooms that have no previous history of insecticide treatments, but have live bed bug infestations were used. Mean (±SE) room size was 11.6 ± 0.2 m^2^. The rooms were left empty for a mean (±SE) of 10 ± 3 days before the treatment started. Prior to desiccant dust application, all skirting in the rooms was loosened. Furniture and personal belongings were removed and subsequently frozen at temperatures below minus 25 °C for a mean (±SE) of 10 ± 1 days. A thin coating of Syloid desiccant dust was applied precisely at the concentration of 1.0 g/m^2^ on the linoleum floor of each room using an exacticide duster (Technicide, California, USA). The dust was focused towards possible hiding places and movement areas of bed bugs. The room was left undisturbed for 7 days. Five of the 11 infested rooms used in the experiment received CO_2_ stimulation, whereas six did not. Untreated controls were not used as we did not want to impart unnecessary stress to the students moving back into control dormitories. CO_2_ was introduced on a daily basis between 1400 h and 2100 h by placing a 600 g block of dry ice directly on the floor at the position of the removed bed. The block of dry ice was allowed to evaporate completely to produce CO_2_ gas for approximately one and a half hours per day. In total, each room received 6 such CO_2_ release events before the desiccant dust treatment was terminated. Temperature and humidity were monitored using Tiny Tag data loggers. Temperature ranged from 18 to 23 °C and relative humidity ranged from 40 to 60 %. After treatment, the desiccant dust was completely removed by vacuuming and cleaning before bringing back the furniture and allowing the students to move back in. All returned beds were covered with protective mattress encasement (Protect-a-bed, Wheeling, IL, USA) to ease detection of newly established bed bug infestations. The complete handling of furniture, removal of skirting, vacuuming and application of desiccant dust took a mean (±SE) of 1.8 ± 0.1 h, in which time it took to introduce the dry ice blocks were not counted.

To assess the level of infestation and to ensure equal infestation levels before the field experiments, we used a time-dependent standardized sampling protocol. Fecal spots on the bedframe were counted for one minute and living bed bugs were collected by vacuuming both the room and bed for 5 min each. Living bed bugs were also collected with 4 Climb-up interceptor traps (Susan McKnight Inc., Memphis, USA) during the desiccant dust treatment, and we collected and counted all the dead bed bugs found in the room after the treatment.

After treatment, the rooms were occupied again. Students were told not to remove the mattress encasement, but no further instructions were given to the student moving back in. After 10–12 weeks, the room was inspected thoroughly with special focus on the bed. If living bed bugs or fecal spots were found on the mattress encasement, then the treatment was scored as a failure, and if no signs of new bed bug infestation could be found, then it was scored a success.

### Statistical analyses

The data were analyzed using SigmaPlot 12.3 (Systat Software, San Jose, CA, USA) and JMP Pro 11.1.1 (SAS Institute, Cary, NC, USA). The data were checked for normality, and pairwise comparisons were performed using t-tests, paired t-tests or Fisher’s exact test between the treatments. If tests for normality failed, we used the nonparametric alternatives, Wilcoxon signed-rank and Mann‐Whitney rank sum test. The level of significance was set at 0.05. We used the Kaplan–Meier product-limit method with the log-rank test between groups to investigate survival.

## Results

### Dose–response of desiccant dusts

**Fig. 2:**
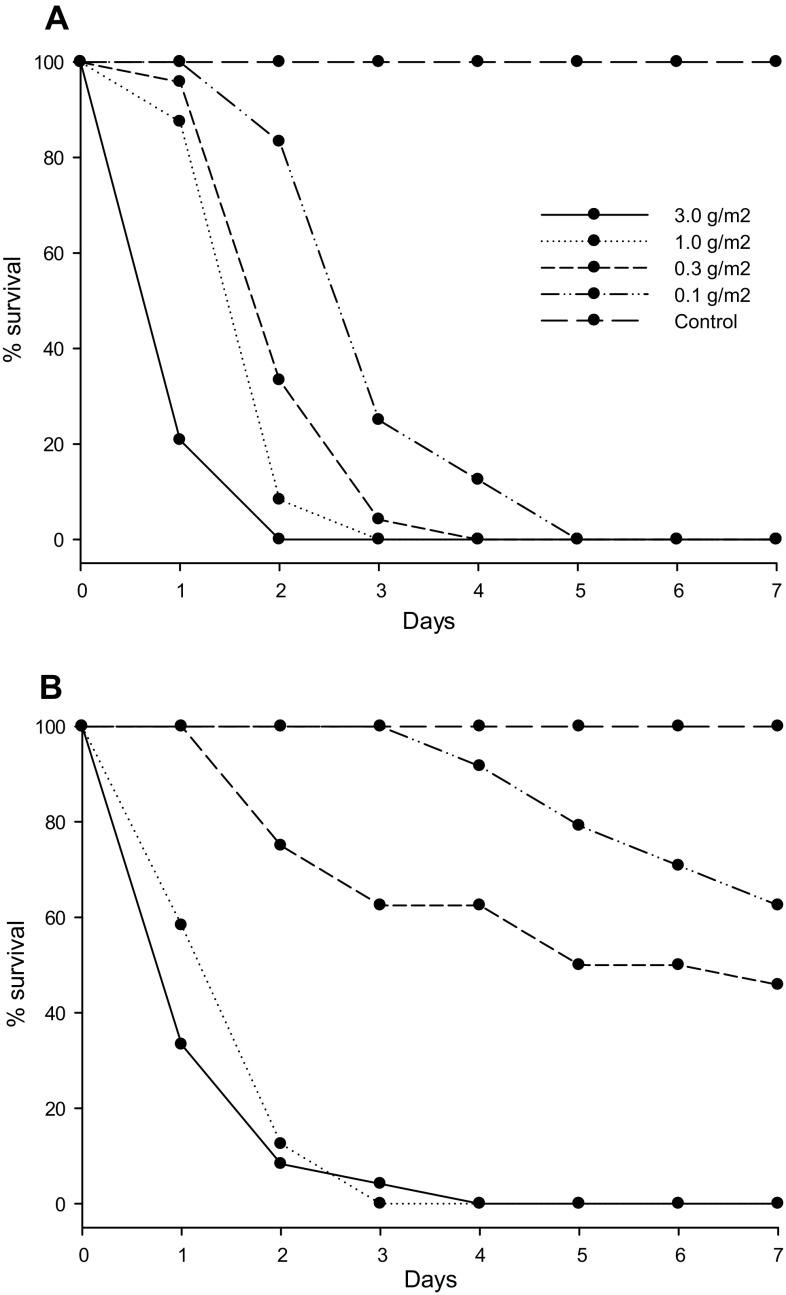
Survival of *Cimex lectularius* in Petri dishes treated with various application rates of **a** Syloid and **b** diatomaceous earth

All desiccant dust treatments induced a significant mortality compared to the control (Kaplan‐Meier log‐rank test—only test for lowest mortality shown: 0.1 g/m^2^ diatomaceous earth vs. Control, *χ* ^*2*^ = 19.82, *P* < 0.001). Syloid applied at 0.1 g/m^2^ killed all bed bugs in five days, and increasing the application rate significantly reduced the time to achieve 100 % mortality (Fig. 2(a), Kaplan–Meier log‐rank tests: 3.0 vs. 1.0 g/m^2^, *χ* ^*2*^ = 21.04, *P* < 0.001; 1.0 vs. 0.3 g/m^2^, *χ* ^*2*^ = 5.14, *P* = 0.023; 0.3 vs. 0.1 g/m^2^, *χ* ^*2*^ = 12.20, *P* < 0.001). Diatomaceous earth was less effective in killing bed bugs, except for the higher application rates of 3.0 and 1.0 g/m^2^ that caused 100 % mortality (Kaplan–Meier log‐rank test: 3.0 g/m^2^—Syloid vs. diatomaceous earth, *χ* ^*2*^ = 1.80, *P* = 0.179; 1.0 g/m^2^—Syloid vs. diatomaceous earth, *χ* ^*2*^ = 1.80, *P* = 0.180). The application rate of 0.3 g/m^2^ produced 50 % mortality in 5 days, while 0.1 g/m^2^ killed less than 50 % within the 7 days of observation (Fig. 2b).

### Activity during CO_2_ stimulation in arena experiments

**Fig. 3:**
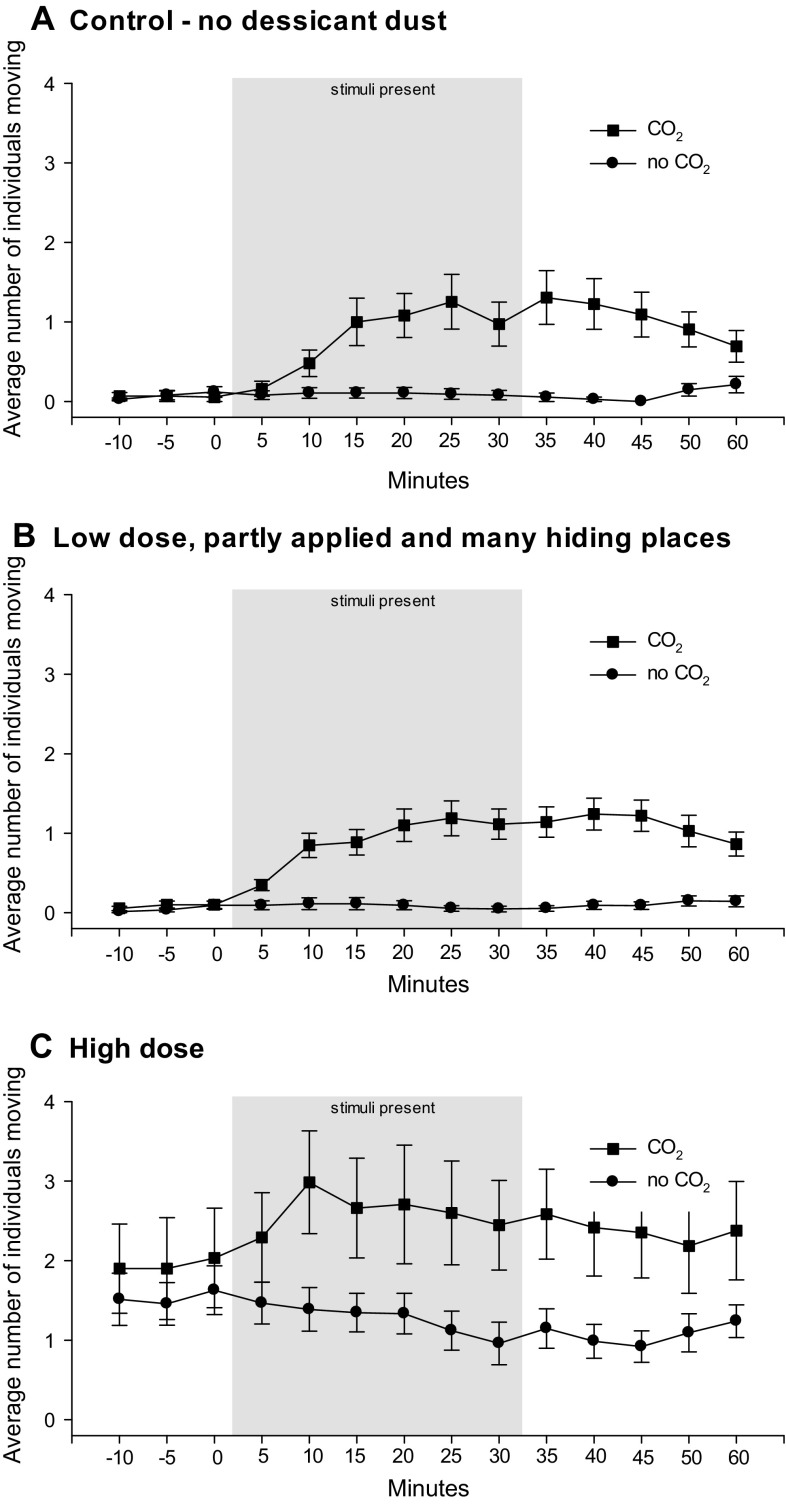
Mean ±SE number of active *Cimex lectularius* in bed bug arenas before, during and after CO_2_ gas stimulation period or no CO_2_ gas stimulation in **a** control treatment with no Syloid desiccant dust, **b** partially applied with a low application rate of Syloid desiccant dust dose and with many potentially new harborages and **c** full application of Syloid desiccant dust with a high application rate

The bed bugs increased their activity during the CO_2_ gas release in all experiments (Fig. 3, paired t-tests, Control: *t* = 7.3, *n* = 11, *P* < 0.001, Low dose: *Z* = 2.9, *n* = 11, *P* < 0.001, High dose: *t* = 19.6, *n* = 11, *P* < 0.001). However, the stimulation only activated a small proportion of the bed bugs, and only 0.0–4.8 individuals moved simultaneously within a 5-min period. With high concentration of desiccant dust (Fig. 3c), general activity before stimulation was higher than with low concentration of desiccant dust (Mann–Whitney rank sum test, Control vs. High concentration: *T* = 21.0, *n* = 6, *P* = 0.002, Low vs. High concentration: *T* = 21.0, *n* = 6, *P* = 0.002), and activity during stimulation ranged from 0.8 to 8.6 individuals moving within a 5-min period.

### Mortality in arena experiments

**Fig. 4:**
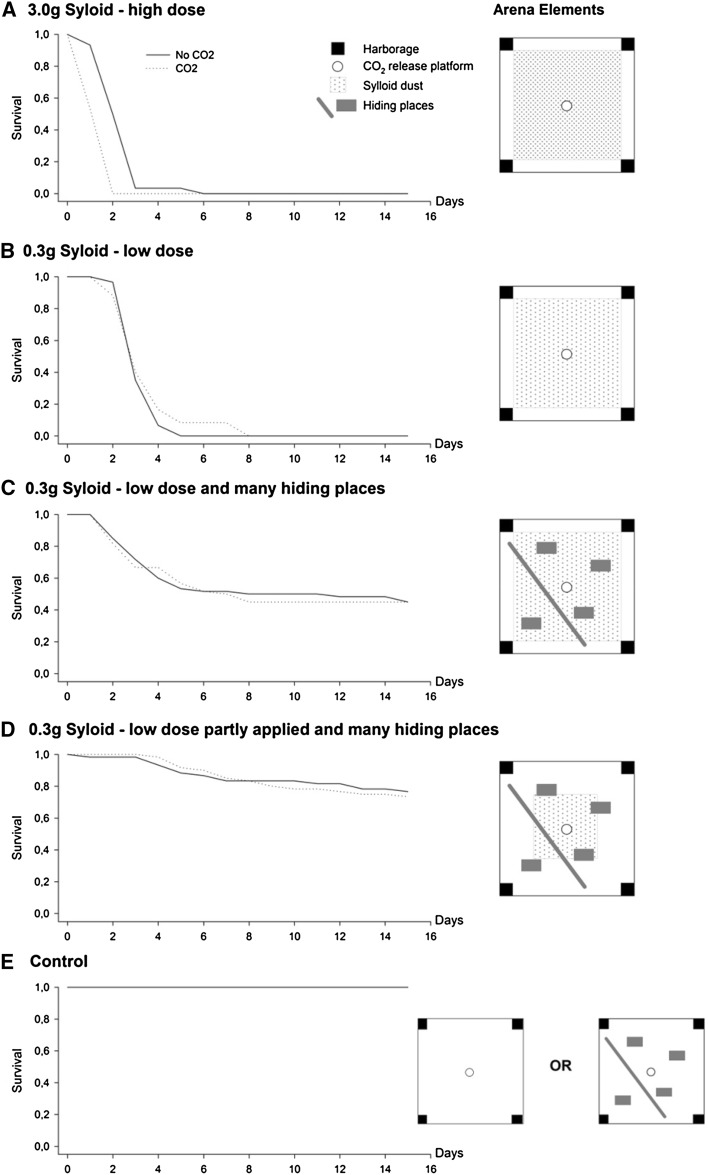
Survival of *Cimex lectularius* in bed bug arenas with different Syloid doses, arena elements and with or without CO_2_

At the highest Syloid concentration, the bed bugs without CO_2_ gas released survived significantly longer than the bed bugs with the CO_2_ released (Kaplan–Meier Log-rank tests; Fig. 4a: *χ* ^*2*^ = 24.64, *P* < 0.001). For other Syloid concentrations, bed bugs showed similar survival regardless of CO_2_ release (Kaplan–Meier Log-rank tests; Fig. 4b: *χ* ^*2*^ = 1.54, *P* = 0.215, Fig. 4c: *χ* ^*2*^ = 0.35, *P* = 0.852 and Fig. 4d: *χ* ^*2*^ = 0.15, *P* = 0.700). We pooled the data across the CO_2_ treatment to test for effects of desiccant dust concentration and availability of potential new harborages. The high-concentration treatment caused 100 % mortality after 2–6 days (Fig. 4a). This was significantly faster than in the low-concentration treatment, which needed 5–8 days to reach 100 % mortality (Kaplan–Meier log-rank test; High vs. Low concentration: *χ* ^*2*^ = 99.97, *P* < 0.001). Introduction of potential new harborages into the arena, while keeping the concentration of desiccant dust low, significantly reduced mortality to approximately 45 % after 14 days (Kaplan–Meier log-rank test; No vs. New potential harborages: *χ* ^*2*^ = 77.13, *P* < 0.001). Keeping the number of new potential harborages constant and reducing the application of desiccant dust by partial treatment resulted in even less mortality, significantly reducing it to approximately 25 % within 14 days (Kaplan–Meier log-rank test; Full vs. Partly applied desiccant dust: *χ* ^*2*^ = 27.22, *P* < 0.001).

### Field experiment

In total, an average (±SE) of 76 ± 25 bed bugs and 108 ± 20 fecal spots were detected per room. All five infestations treated with desiccant dust in combination with CO_2_ succeeded in eradicating bed bugs, whereas all six infestations treated with only desiccant dust failed to do so. The effect of CO_2_ on eradication success was significant (Fisher’s exact test; *p* = 0.002) and prior to treatment the infestation levels in rooms given dust treatment only was similar to infestation levels in rooms given dust treatment in combination with CO_2_ activation (bed bugs collected: Mann–Whitney rank sum test; *T* = 26.0 *n* = 5/6 *P* = 0.537, fecal spots counted; *t* test; *t* = 0.203, *n* = 5/6, *P* = 0.844).

## Discussion

Bed bugs have remarkable abilities to handle environmental stress (Benoit et al. 2009a; Benoit 2011; Rukke et al. 2014; DeVries et al. 2015), which is partly a benefit of their ability to conserve water through their tough cuticle (Benoit et al. 2007). Their cuticle is however vulnerable, and we showed that targeting the exoskeleton with desiccant dust can be a strategy for eradication of bed bug populations. Very little is known about durability in the bed bug integument (Koganemaru et al. 2013; Lilly et al. 2016), but survivorship appears to be strongly dependent on the full functionality of this structure. Generally, the integument consists of the basal lamina, epidermis, endocuticle, exocuticle, and finally epicuticle, and the structural balance between these layers determines the mechanical properties (Chapman 2013). It is not known how the desiccant dust treatments cause mortality in bed bugs, but it is generally believed that diatomaceous earth provides an abrasive function, whereas Syloid breaks down the cuticle by absorbing some elements of the wax layer (Ebeling 1971; Shah and Khan 2014). Increased knowledge of formulations and adhesive properties of desiccant dusts as well as details about the bed bug integument structure may promote the development of more effective desiccant dusts. Regardless of the mechanism of the desiccant dusts, we showed that rapid mortality can be achieved with either Syloid or diatomaceous earth and that Syloid was the more effective of the two. Previous studies have also shown that properly formulated and applied desiccant dusts at the right concentration may kill bed bugs in less than a week (Benoit et al. 2009b; Anderson and Cowles 2012; Akhtar and Isman 2016).

The efficacy at lower concentrations advocates that Syloid should be favored over diatomaceous earth as a killing agent for bed bugs. Both have relatively large particle size and low toxicity to humans (Jaganathan and Godin 2012), but they will have a negative impact on the skin, airways and mucous membranes by causing some desiccation and irritation. However, as the short- and long-term effects of human exposure to Si-based particles are not completely understood (Jaganathan and Godin 2012), using the lowest possible effective concentration is of importance. Small amounts will ease complete removal of dust after treatment to reduce any potential human health risk. The Petri dish and the arena experiments indicate that application rates as small as 0.1–0.3 g/m^2^ are sufficient. However, the reduced effect by adding hiding places or only doing a partial cover of surfaces indicated that higher doses are needed in actual control situations. We used Syloid at 1.0 g/m^2^ in our field experiment, but this was combined with careful distribution and uncovering of hiding places. This approach was labor intensive and future control situations may benefit from somewhat larger doses.

We observed a variable effect of CO_2_ activation. Mortality did only increase with the stimulus in one out of four experiments in the laboratory, while it appeared to be crucial for the success of eradication in the field. This difference may be explained by the short periods of the laboratory activation leading only to a low proportion of bed bugs responding as compared to the more intense activation in the field. Bed bugs will normally also engage in some spontaneous questing during the night (Romero et al. 2010; Aak et al. 2014; Cooper et al. 2015), and it is likely that this general activity in the arena experiment exceeds the increased activity from the stimulation making the two treatments more or less equal. In the laboratory experiment, the CO_2_ gas releasing period or the concentration of the gas may not have been enough to increase the bed bug mortality. The blocks of dry ice used in the intense stimulation in the field study weighed 600 g and evaporated in approximately 1.5 h. This is a release rate of 3300 mg/min, which is about 5- to 10-fold the natural release from a resting human and also 10-fold the dose used in the arena. This boost of host signal may have contributed to enhance mortality. It is also possible that such stimulation will have even stronger effects if it takes place during the night. It is tempting to solely focus on the successful use of the CO_2_ gas, but it is equally important to highlight the 100 % failure of the dust treatments when tested in the field without CO_2_ activation. This means that desiccant dust may probably demand support from other control efforts in IPM solutions (Wang et al. 2009a; Wang et al. 2013) and if used as a single control method will need the benefit from combination with an activating stimulus (Benoit et al. 2009b).

Occurrence of potential new harborages decreased killing efficacy in the laboratory experiments. As we cleaned the arenas between the experiments, the aggregation pheromones that normally keep bed bugs inside harborages (Siljander et al. 2008; Olson et al. 2009; Weeks et al. 2011; Weeks et al. 2013; Gries et al. 2015) would be removed. Thus, in a natural situation, hiding places might be even more important in restricting bed bug movement. This is an additional argument for mimicking presence of a host with release of CO_2_ gas in a bed bug control situation. In our field experiment, we did a thorough job at detecting and revealing harborages. However, it is highly unlikely that we uncovered all bed bug harborages, and the use of CO_2_ gas probably lured out the hiding individuals that were not detected to increase exposure to the desiccant dust. Several other studies have also shown the effect of CO_2_ on activity and trap catches in both laboratory and the field (Anderson et al. 2009; Wang et al. 2009b; Suchy and Lewis 2011; Singh et al. 2012; Singh et al. 2013; Aak et al. 2014; Singh et al. 2015a; Singh et al. 2015b), but none has attempted to use host signals as a tool to improve killing efficacy. The use of an activating stimulant bears resemblance to traditional attract-and-kill strategies (El-Sayed et al. 2009) and appears to be very effective in the confined and relatively small bed bug habitats. A host mimicking signal may also be further improved by adding additional host odors (Weeks et al. 2010; Harraca et al. 2012) or by adjusting release rates to more closely resemble human presence (Singh et al. 2013; Singh et al. 2015b). The dry ice stimulant may also be replaced with other CO_2_-delivering devices such as sugar-yeast solutions or CO_2_ pressure tanks to produce similar effects (Anderson et al. 2009; Singh et al. 2012; Singh et al. 2015a; Singh et al. 2015b). Such a solution is also more practical as the stimulant can be prepared on site to produce a lasting stimulation.

In the field approach, the rooms were left empty prior to desiccant dust application. This allows bed bugs to get hungry and more susceptible to CO_2_ stimulation (Aak et al. 2014) and to let recently deposited eggs hatch. This wait-before-kill approach followed by the attract-and-kill strategy may be a somewhat overlooked feature of bed bug control. If detection of a bed bug infestation leads to immediate actions, or if people use the room until treatment begins, it is likely that a quite large proportion of the population is fully engorged and remains passive and concealed in harborages for several days (Wintle and Reinhardt 2008; Aak et al. 2014). Additionally, blood feeding and engorgement allow recovery and induce resilience to pesticides (Feldlaufer et al. 2013; Choe and Campbell 2014) including desiccant dust (Singh et al. 2016). Hungry individuals may consequently be an easier target. Most egg laying also takes place during the first week after feeding and at optimal temperatures, these eggs will not hatch for another 4–5 days (Suwannayod et al. 2010; Polanco et al. 2011). This means that, at room temperature, at least 14 days are needed before one can expect all individuals in a population to be susceptible to the treatment. The time needed to produce hungry bed bugs and ensure hatching of all eggs can possibly be reduced by increased metabolism at elevated temperatures (DeVries et al. 2013). Sufficient time will increase bed bug responsiveness to host signals (Aak et al. 2014) and temperatures above bed bug optimum might induce some level of thermal stress (Benoit 2011; Rukke et al. 2014) to add to the effect of the total control effort.

Bed bug field studies are rare because completely controlled experiments are difficult to achieve, and assigning control or partial treatment to participants is ethically arduous. Most studies of new eradication techniques are therefore often tested as part of an IPM program (Wang et al. 2009a; Wang et al. 2013), making isolation of effects difficult and quantification even harder. In our study, we took great care in isolating the effect of desiccant dust from the combined effect of desiccant dust and CO_2_ activation. We showed that use of CO_2_ as a lure mimicking human presence in combination with desiccant dust appears to be a promising option for bed bug control. The cost of adding CO_2_ gas during treatment is low and can easily be incorporated in traditional IPM strategies for bed bug control. It might also be combined with new potential insecticides such as fungus (Barbarin et al. 2012) or PBH-synergized pyrethroid (Hardstone et al. 2015).

## Author contribution

AA, ER, BAR, and TB conceived and designed the research. AA, ER, and BAR conducted laboratory experiments. ER conducted field experiments. AA, ER, and BAR processed and analyzed the data. AA, TB, and ER wrote the manuscript. All authors contributed to the writing process and approved the manuscript.
